# Novel TLR2 agonist Amuc_C derived from *Akkermansia muciniphila* exhibits potent anti-tumor activity in colorectal cancers

**DOI:** 10.1080/19768354.2025.2578019

**Published:** 2025-10-27

**Authors:** Liang Chi, Chiao-Hsu Ke, Hsin-Yi Wu, I.-Li Liu, Chih-Hung Huang, Chen-Si Lin

**Affiliations:** a Department of Veterinary Medicine, School of Veterinary Medicine, National Taiwan University, Taipei, Taiwan; b Instrumentation Center, National Taiwan University, Taipei, Taiwan; c Institute of Veterinary Clinical Science, School of Veterinary Medicine, National Taiwan University, Taipei, Taiwan; d Department of Chemical Engineering and Biotechnology, Institute of Chemical Engineering, National Taipei University of Technology, Taipei, Taiwan

**Keywords:** Toll-like receptor 2 agonist, *Akkermansia muciniphila*, Amuc_1100 C-terminal, immunomodulatory agents, translational medicine

## Abstract

Colorectal cancer (CRC) is a challenging disease. Recent studies have gradually emphasized the development of novel immunotherapies rather than traditional treatments. Toll-like receptor (TLR) agonists are critical in innate immune responses to orchestrate anti-tumor efficacies, which are attributed to their aptitude to stimulate antigen-presenting cells (APCs) and thus activate tumor-specific T cells. Although several TLR agonists have been proposed for treating tumors, their therapeutic efficacy remains controversial. Therefore, the current study aimed to develop a novel TLR2 agonist, Amuc_1100 C-terminal (Amuc_C), a purified membrane protein from *Akkermansia muciniphila* (*A. muciniphila*), and evaluate its anti-tumor properties. Herein, a murine CRC model, CT26, was employed. Tumor-bearing mice received intertumoral treatment with Amuc_C. The anti-tumor effects were determined by flow cytometry, cytokine enzyme-linked immunosorbent assay (ELISA), and immunofluorescence assays. Liquid chromatography-tandem mass spectrometry (LC-MS/MS) was then employed to uncover the potent mechanisms. Amuc_C significantly increased the amounts of tumor-infiltrating lymphocytes and systemic immune cells, especially cytotoxic T cells, M1 macrophages, and type 1 dendritic cells. Furthermore, Amuc_C triggered IL-1β, TNF-α, and IFN-γ productions, significantly decreasing tumor growth, and prolonged overall survival. The immunotherapeutic mechanisms revealed by proteomics data were related to the activation of immune responses, the induction of cell cycle arrest, and the inhibition of cell proliferative signaling pathways. In summary, the current study has demonstrated that administration of Amuc_C improves the APCs and escalates adaptive anti-tumor immunity. With the demand for effective anti-tumor treatments, our results provide a compelling proof-of-concept of a TLR2 agonist for cancer immunotherapy.

## 1. Introduction

The microenvironment colonized by a tremendous number of microorganisms is known as microbiome (Sender et al. 2016). The roles of microbiome that regulated the innate and adaptive immune systems have been widely discussed. For example, they regulated immune system development and immune homeostasis. The well-studied communication of host-microbiota is the intestinal mucosa. The intestinal mucosa concurrently upholds protective immunity against pathogens, and the traits of immune tolerance toward a massive and ever-shifting population of benign microbes are sustained (Zheng et al. 2020). While organized around hyperglycosylated MUC2, the mucus barrier does more than physically shield. It also suppresses immune activation against gut antigens by programming intestinal dendritic cells (DCs) toward anti-inflammatory responses (Shan et al. 2013). Therefore, it is believed that the perturbation of the gut microbiome might result in a variety of immune-related gastrointestinal diseases, such as inflammatory bowel disease (IBD) (Zhang et al. 2017) and even malignancy (Gopalakrishnan et al. 2018). The correlation between the gut microbiome and the inhibition of colorectal cancer (CRC) growth has also been found (Tilg et al. 2018; Wong and Yu 2019), emphasizing the importance to maintain gut health in the control of immune and malignant diseases. One of the specific bacteria is *Akkermansia muciniphila* (*A. muciniphila*), which is a gram-negative anaerobic bacterium that is a component of the commensal microbiota in humans (Luo et al. 2022) and even companion animals (Garcia-Mazcorro et al. 2020; Giaretta et al. 2020).

As a commensal microbiota in humans and different species, the characteristics of *A. muciniphila* have been studied for decades. An abundance of *A. muciniphila* has been reported to correlate with IBD in humans, and the abundant presence of *A. muciniphila* is considered a favorable prognostic indicator for CRCs (Earley et al. 2019; Gu et al. 2021; Gubernatorova et al. 2023). Immunomodulatory and anti-tumorigenesis functions against CRCs have also been reported, especially the activation of toll-like receptor 2 (TLR2)/nucleotide-binding oligomerization domain-leucine rich repeats-containing receptor 3 (NLRP3)-mediated M1 tumor-associated macrophages, inhibiting tryptophan metabolism through the AhR/β-catenin signaling pathway (Fan et al. 2021b; Zhang et al. 2023). Several proteins of *A. muciniphila* have been isolated, and each has different properties and mechanisms that suppress tumor growth (Wang et al. 2020a). An isolated outer membrane protein, Amuc_1100, was found to be a potent TLR2 agonist (Wang et al. 2020a; Si et al. 2022). Amuc_1100 is involved in the development of pili, promoting interactions with TLR2 (Ottman et al. 2017). This attribute positions it as a noteworthy contender for drug development initiatives. Another feature of Amuc_1100 is that it retains its biological activity even after pasteurization at 70 °C for 30 min (Eslami and Yousefi 2022; Si et al. 2022). Furthermore, anti-tumor mechanisms such as induction of cytotoxic T lymphocytes (CTLs) and tumor necrosis factor-α (TNF-α) secretion, downregulation of Ki-67s, and decreasing PD-1+ T cells have also been identified (Dutta and Lim 2020; Derosa et al. 2022). Therefore, Amuc_1100 is a potentially promising anti-cancer agent, and further studies are being conducted.

In addition to the cytotoxic immune responses, improving the antigen presentation ability is critical in treating CRCs with a low tumor mutation burden (Fan et al. 2021a). Given the capacity of Amuc_1100 to activate the immune system and other signaling pathways to inhibit tumor growth, we decided to investigate this therapeutic compound further. Although the anti-tumor effects of Amuc_1100 have been proposed, its relatively large molecular size, due to its being a membrane protein, may result in lower absorption efficiency in organisms compared to smaller peptides. Furthermore, Amuc_1100 requires freezing for preservation, but the freezing process could damage its structure. Therefore, it is necessary to confirm whether the non-intact structure of Amuc_1100 retains the same bioactivity and anti-cancer effects. Herein, we developed a new compound, Amuc_1100 C-terminal (Amuc_C), to overcome these difficulties. This study demonstrates that Amuc_C exhibits effective anti-cancer properties and explores its immunomodulatory and anti-cancer mechanisms.

## 2. Materials and methods

### 2.1. Cell culture and determination of Amuc_C

RAW264.7 and DC2.4 cell lines were kindly provided by Dr. Yu-Ling Lin (Agricultural Biotechnology Research Center, Academia Sinica, Taipei, Taiwan), Prof. Ming-Thau Sheu and Shyr-Yi Lin (Taipei Medical University, Taipei, Taiwan), respectively. RAW264.7 was maintained in DMEM (Corning, Cat. No. 10-013-CM) with 10% fetal bovine serum (FBS, Thermo Fisher Scientific, Waltham, MA, USA, Cat. No. 10100147) and 1% penicillin–streptomycin (Corning, Cat. No. 30-002-CI) in an incubator at 37 °C and 5% CO_2_. DC2.4 was maintained in RPMI-1640 (Sigma–Aldrich, St. Louis, MO, USA, Cat. No. R0883) supplemented with 1X L-Glutamine (Millipore, Cat. No. TMS-002-C), 1X non-essential amino acids (Millipore, Cat. No. TMS-001-C), 1X HEPES Buffer Solution (Millipore, Cat. No. TMS003-C) and 0.0054X β-Mercaptoethanol (Millipore, Cat. No. ES-007-E), 10% fetal bovine serum (Thermo Fisher Scientific, Cat. No. 10100147) and 1% penicillin–streptomycin (Corning, Cat. No. 30-002-CI). CT26.WT was purchased from America Type Culture Collection (ATCC® No. CRL-2638™) and cultured with RPMI-1640 (Corning, Cat. No. 10-040-CM) with 10% fetal bovine serum (Thermo Fisher Scientific, Cat. No. 10100147) and 1% penicillin–streptomycin (Corning, Cat. No. 30-002-CI). The LookOut® Mycoplasma PCR Detection Kit (Merck, Darmstadt, Germany, Cat. No. MP0040A) was used to regularly screen Mycoplasma contamination.

**Figure 1.**
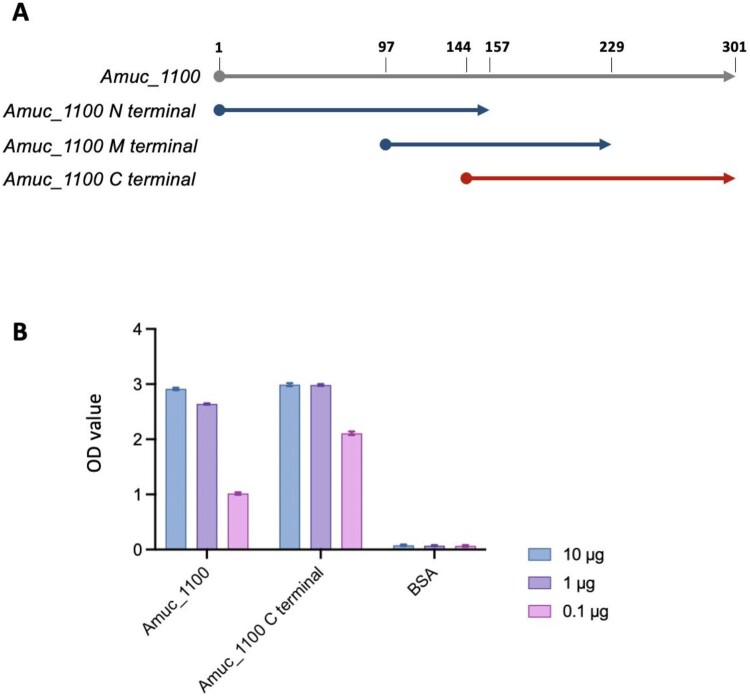
Determination of the functional structure in Amuc_1100 that could trigger TLR2 signaling transduction. (A) Definitions of the N-terminal, middle, and C-terminal parts depending on sequence length for Amuc_1100. (B) TLR2 signaling induced by Amuc_1100 and C-terminal of Amuc_1100* detected by SEAP reporter gene assay. Samples and controls are tested in duplicate on recombinant HEK-293 cell lines. These cell lines functionally over-express human TLR2 as well as a reporter gene, which is a SEAP. The production of this reporter gene is driven by an NF-ĸB inducible promoter. Activation results are given as optical density values. SEAP, secreted alkaline phosphatase.

Numerous studies have reported that the full-length of Amuc_1100 is needed to activate TLR2 agonistic effects. To understand which segment of the structure in Amuc_1100 could trigger TLR2 signaling transduction, we defined the N-terminal (1–157), middle (97–229), and C-terminal (144–301) parts depending on sequence length for Amuc_1100 (Figure 1(A)). We evaluated the agonistic effect of the C-terminal and the remaining portions on HEK293-TLR cell lines by secreted alkaline phosphatase (SEAP) reporter gene assay. The results revealed that only the C-terminal could activate TLR2, whereas other parts of Amuc_1100 failed to provoke TLR2 activities. These results suggested that Amuc_C was the central functional region that could provoke TLR2 signaling of Amuc_1100 (Figure 1(B)).

### 2.2. Cell viability assay

RAW264.7 and DC2.4 cells were seeded into 96-well plates at the seeding density of 5 × 10^4^ cells/well and cultured with medium supplemented with 1% FBS containing different concentrations of Amuc_C (2.5, 5, and 10 μg/ml) in an incubator at 37 °C for 24 h. After 24 h, CCK-8 (TargetMol, Cat. No. C0005) was added and cultured in the incubator for 1 h, and the absorbance values were measured with an ELISA reader (SpectraMax® M5 Microplate Reader, San Jose, CA, USA) at a wavelength of 450 nm.

### 2.3. Animal experiments

BALB/c mice were inoculated subcutaneously with CT26 CRC cells (3 × 10^5^ cells per mouse) on day 0, and the treatment started when the tumor volume was in the range of 50–100 mm^3^. On day 14, CT26-bearing mice were administered 20 μg (1 mg/kg) of Amuc_C or the same volume of PBS once every three days via intramuscular injection, and tumor volumes were also measured every 3 days until the endpoint of the experiment. After 5 times of treatments, all mice were sacrificed on day 29 to collect tumors and spleens for further experiments. For survival analysis, mortality was based on mouse removal from the study once tumor volumes exceeding 1000 mm^3^ or ulcerations were found at tumor sites. All animal experiments were approved by the Institutional Animal Care and Use Committee, National Taiwan University (IACUC Approval No. NTU-111-EL-00039).

### 2.4. Splenocyte isolation and flow cytometry analysis

Spleens were pressed by the end of a plunger and filtered with 70 μm cell strainers, and the erythrocytes were lysed using sterile RBC lysis buffer (Thermo Fisher Scientific, Cat. No. 00-4333-57). For blocking of Fc receptors, 1 × 10^6^ of RAW264.7 cells, DC2.4 cells, and isolated splenocytes were first stained with anti-mouse CD16/32 (TruStain FcX™ PLUS Antibody; BioLegend, Cat. No. 156603) for 10 min at 4 °C. The following monoclonal antibodies were used to assess the phenotypes of T lymphocytes, macrophages, and dendritic cells: PE/Cyanine7 anti-mouse CD45 (BioLegend, clone 30-F11, Cat. No. 103114), FITC anti-mouse Ly-6C (BioLegend, clone HK1.4, Cat. No. 128006), rat anti-Mouse CD3: FITC (Bio–Rad, Hercules, CA, USA, clone KT3, Cat. No. MCA500F), rat anti-Mouse CD4: RPE (Bio–Rad, clone RM4-5, Cat. No. MCA2691PE), rat anti-Mouse CD8: Alexa Fluor® 647 (Bio–-Rad, clone YTS169.4, Cat. No. MCA1768A647), PE anti-mouse F4/80 (BioLegend, clone BM8, Cat. No. 123110), APC/Cyanine7 anti-mouse/human CD11b (BioLegend, clone M1/70, Cat. No. 101226), PerCP/Cyanine5.5 anti-mouse I-A/I-E (BioLegend, clone M5/114.15.2, Cat. No. 107626), APC anti-mouse CD11c (BioLegend, clone N418, Cat. No. 117310), and PE anti-mouse CD103 (BioLegend, clone 2E7, Cat. No. 121406). Cells were incubated with corresponding conjugated antibodies for 30 min at 4 °C and analyzed with a BD LSRFortessa Cell Analyzer (BD Biosciences, San Jose, CA, USA). The flow cytometric data were then analyzed in the FlowJo v10 software (BD Biosciences).

### 2.5. Immunofluorescence staining

Immunofluorescence staining for CD3 (iReal, Cat. No. IR252-1), CD4 (Bioworld, Cat. No. BS6982), CD8 (Genetex, Cat. No. GTX74776), F4/80 (Abcam, Cat. No. ab6640), CD86 (Bioworld, Cat. No. bs0099 m), iNOS (Bioss, Cat. No. bs-2072R), CD206 (ACE, Cat. No. A21142PI), Arginase-1 (Cell Signaling, Cat. No. 93668), CD11c (ABcloncal, Cat. No. A1508), and MHCII (Bioworld, Cat. No. BD90647) antigens was conducted with tools of a molecular fluorescence multiple stain kit (Biotna, Cat. No. TATS01F) following the manufacturer’s instructions. The dilution factors and incubation times of each primary antibody were 400x (1 h), 800x (2 h), 400x (2 h), 1000x (16 h), 500x (16 h), 200x (2 h), 200x (16 h), 100x (2 h), 1000x (1 h), and 100x (1 h), respectively. All primary antibodies were incubated at room temperature. Sections were then stained with the following secondary antibodies: Goat anti-Rabbit IgG (H + L)−488 (Biotna, Cat. No. TAFB02-488), Goat anti-Mouse IgG (H + L)−594 (Biotna, Cat. No. TAFB02-594), and Goat anti-Rabbit IgG-iFluor-680 (Biotna, Cat. No. TAFB02-680) at 4 °C for 30 min, and DAPI was used for counterstaining. Whole slide imaging was digitalized by an Olympus VS120 Virtual Slide Microscope (Olympus Corporation, Japan) at 20×, and Olympus OlyVIA software was used to review the slide images. All procedures were conducted by Li-Tzung Biotechnology INC (Kaohsiung, Taiwan). CD8, CD86, CD206, and CD11c single-color images were quantified randomly in five fields at 20x magnification.

### 2.6. Cytokine ELISA

Splenocytes (4 × 10^6^ cells) from untreated tumor-bearing mice were seeded into a 12-well tissue culture plate in different wells. The splenocytes were then divided into the control group and the Amuc_C-treated group. The control group was cultured with RPMI-1640 with 20% FBS only, and the Amuc_C group was treated with 10 μg/ml Amuc_C for 72 h respectively. For the cytokine detection of the combination therapy test, splenocytes were incubated with RPMI-1640 supplemented with 20% FBS for 72 h. Finally, the supernatant was collected for cytokine ELISA analysis. The IL-1 beta Mouse ELISA Kit (Invitrogen, Cat. No. BMS6002), TNF alpha Mouse Uncoated ELISA Kit (Invitrogen, Cat. No. 88-7324-88), and IFN gamma Mouse Uncoated ELISA Kit (Invitrogen, Cat. No. 88-7314-88) were utilized to quantify the concentration of the cytokines following the manufacturer’s instructions. The OD values were measured with an ELISA reader (SpectraMax® M5 Microplate Reader) at a wavelength of 450 nm.

### 2.7. Protein sample preparation and LC-MS/MS analysis

The sample preparation and LC-MS/MS analysis processes were as previously described (Ke et al. 2022). Briefly, tumor tissues dissected from sacrificed mice were preserved in liquid nitrogen before protein extraction. The frozen tissues were ground to powder by mortar and pestle with an adequate volume of RIPA lysis buffer (Sigma–Aldrich, Catalog No. R0278) and then placed into a -80 °C freezer for 1 h followed by centrifugation to obtain the supernatant, which was the protein of the tumors. The supernatant was precipitated with acetone and reconstituted with 6M urea afterward. Bradford protein assay was performed to quantify the protein concentration with Protein Assay Dye Reagent Concentrate (Bio–Rad, Cat. No. #5000006). A total of 50 μg of each protein sample was submitted for liquid chromatography with tandem mass spectrometry (LC-MS/MS) using an Orbitrap Fusion Lumos Tribrid quadrupole-ion trap–Orbitrap mass spectrometer (Thermo Fisher Scientific). In-solution digestion and desalting were conducted before LC-MS/MS analysis to increase peptide sequence convergence and decrease chemical noise in the mass spectra. To select the differentially expressed proteins (DEPs), master proteins with a false detection rate (FDR) < 1% were chosen, and the log_2_ ratio of all chosen proteins ± one standard deviation (SD) was set as the borderline of upregulation and downregulation.

### 2.8. Bioinformatic analysis

Gene Ontology (GO) enrichment analysis and KEGG (Kyoto Encyclopedia of Genes and Genomes) (Dennis et al. 2003) were performed to find out the gene functions and enrichment pathways of all DEPs by using the Database for Annotation, Visualization and Integrated Discovery (DAVID, https://david.ncifcrf.gov/tools.jsp) (Gene Ontology, C. 2006). The heatmap was generated in Morpheus (https://software.broadinstitute.org/morpheus/) to visualize hierarchical clustering (Ryan et al. 2019). The DEPs were imported to STRING (Search Tool for the Retrieval of Interacting Genes/Proteins) to obtain the protein – protein interaction (PPI) networks, which were imported to Cytoscape_v3.10.1 for further analysis, and several plugins were applied. First, the most important cluster in the PPI network was found by MCODE, and the genes included in this cluster were regarded as hub genes. Next, to understand the gene functions and enrichment pathways against these hub genes, BiNGO and Metascape (https://metascape.org/gp/index.html#/main/step1) were utilized to conduct GO term and KEGG analysis once again. Finally, Ingenuity Pathway Analysis (QIAGEN IPA) was employed to find the possible upstream regulators and canonical pathways.

### 2.9. Statistical analysis

All statistical results were analyzed by GraphPad Prism 8 and are presented as mean ± standard error of the mean (SEM). Unpaired two-tailed Student’s t-test and one way-ANOVA with Tukey test were utilized to assess the significant differences among the treatment groups, and the Mantel–Cox test was used to determine the difference in survival rate. A *p*-value < 0.05 was considered statistically significant.

## 3. Results

### 3.1. Amuc_C activates and induces the secretion of TNF-α of antigen-presenting cells

**Figure 2.**
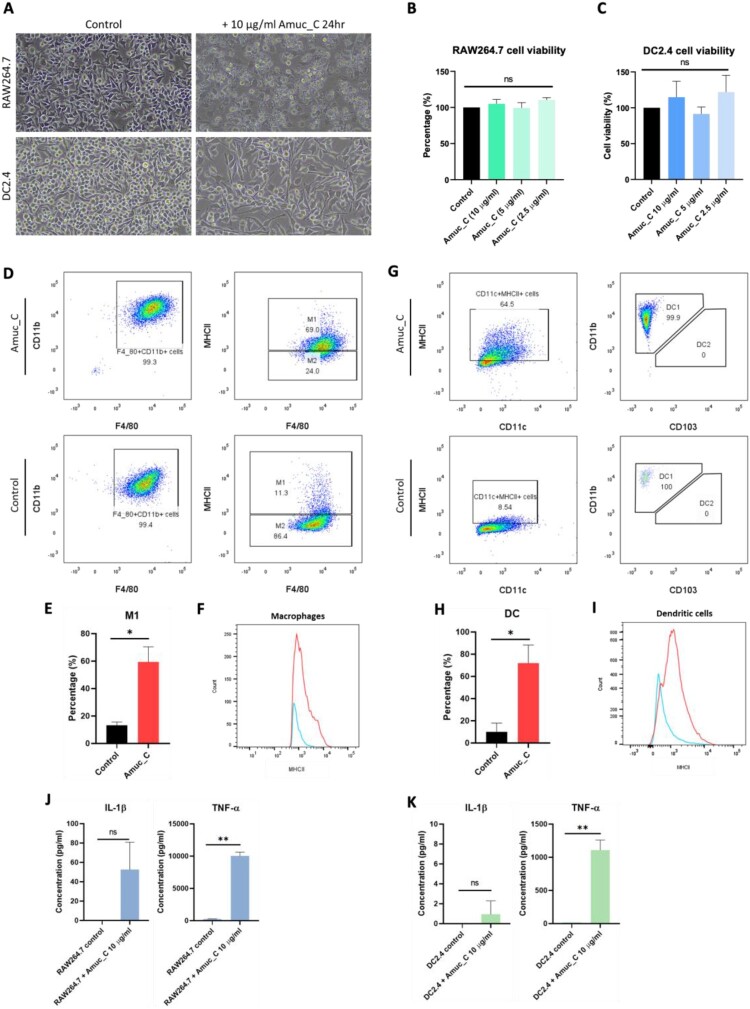
Amuc_C activates antigen-presenting cells and partially induces Th-1 cytokines *in vitro*. (A) The cell morphology of unstimulated and stimulated RAW264.7 and DC2.4. (B) The bar chart of cell viability for Amuc_C-treated RAW264.7 and (C) DC2.4 at different Amuc_C concentrations. (D) Representative flow cytometry dot plots for total macrophages and M1, and (E) bar charts for M1 populations of Amuc_C-treated RAW264.7, (F) MFI for MHCII expression of total macrophages was measured. (G) Representative flow cytometry dot plots for total DCs and DC1 and (H) bar charts for DC1 populations of Amuc_C-treated RAW264.7, (I) MFI for MHCII expression of total DCs was measured. (J) IL-1β and TNF-α in the supernatant of Amuc_C-treated RAW264.7 and DC2.4 were measured by cytokine ELISA. All data are presented as mean ± SEM. Statistical significance was calculated by unpaired two-tailed Student’s t-test to compare two groups (ns, not significant; *, *p* < 0.05; **, *p* < 0.01). DC, dendritic cell; IL-1β, interleukin-1 beta; MHCII, class II of major histocompatibility complex; MFI, Median fluorescent intensity. TNF-α, tumor necrosis factor-alpha.

TLRs are expressed on APCs, and the activation of TLRs can induce the maturation of APCs (Crofts et al. 2023). To investigate the impacts of the novel TLR2 agonist, Amuc_C, on APCs, RAW264.7 and DC2.4 cell lines were treated with 10 μg/ml of Amuc_C. After the 24-hour treatment, RAW264.7 changed from polygonal to rounded shapes, accompanied by numerous vacuoles. DC2.4 cells also displayed increased size and extended dendrites, which indicated that the Amuc_C effectively activated these immune cells (Figure 2(A)). To investigate whether Amuc_C would stimulate the proliferation of APCs, cell viability assays were performed. However, there were no significant effects of Amuc_C on the cell viability of APCs, suggesting that Amuc_C failed to stimulate the proliferation (Figure 2(B,C)). Flow cytometry was then employed to analyze the changes in the phenotypes of the APCs. Notably, increased expression of MHCII was found in both macrophages and dendritic cells. Among these, M1 phenotype macrophages and type 1 dendritic cells were dominant (Figure 2(D–F and G–I)). Furthermore, when RAW264.7 and DC2.4 were stimulated by Amuc_C, increased levels of TNF-α were found, whereas there was no significance of IL-1β after stimulation (Figure 2(J and K)). These results demonstrate that Amuc_C effectively activated macrophages and dendritic cells, and the activated APCs secreted cytokines, especially TNF-α, that might contribute to Th1 inflammatory responses.

### 3.2. Amuc_C inhibits tumor progression without apparent biological toxicity

**Figure 3.**
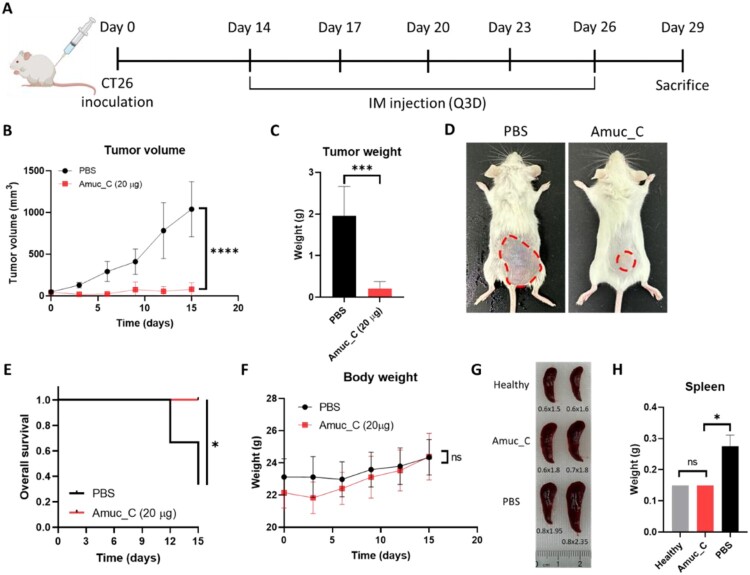
Amuc_C inhibits tumor progression *in vivo* without apparent biological toxicity. (A) A schematic diagram of the animal experiment. BALB/c mice (n = 5) were inoculated subcutaneously with CT26 colorectal cancer cells on day 0, and the treatment started when the tumor volume was between 50–100 mm^3^. On day 14, CT26-bearing mice were administered with 20 μg of Amuc_C or the same volume of PBS once every three days via intramuscular injection, and tumor volumes were also measured every 3 days until the endpoint of the experiment. After 5-time treatments, all mice were sacrificed on day 29 to collect tumors and spleens for further experiments. Mice were sacrificed when the tumor exceeded 1000 mm^3^. (B) Tumor growth and (C) tumor weight of CT26 tumor-bearing mice were measured every three days after treatment. (D) The outward appearance of CT26 tumor treated with PBS or Amuc_C. (E) Overall survival of CT26 tumor-bearing mice treated with PBS or Amuc_C using the Kaplan-Meier method. (F) Body weight of PBS group and Amuc_C group. (G) Spleens from healthy and tumor-bearing mice and (H) spleen weight. All data are presented as mean ± SEM. Statistical significance was calculated by unpaired two-tailed Student’s t-test for the comparisons between the two groups (ns, not significant; *, *p* < 0.05; ***, *p* < 0.001; ****, *p* < 0.0001).

The aforementioned findings suggested that Amuc_C could effectively activate APCs and induce cytokine secretion. However, the anti-tumor effects remained unknown. The schematic diagram of animal experiments is illustrated in Figure 3(A). Amuc_C significantly inhibited the tumor growth rate when mice were administered 20 μg of Amuc_C via intramuscular injection, as compared to the control group receiving PBS (*P* < 0.0001, Figure 3(B)). Consequently, tumors in the Amuc_C-treated group exhibited significantly smaller volumes and lighter weights (Figure 3(C and D)). Mice treated with Amuc_C demonstrated a noticeably longer overall survival time (Figure 3(E)). To assess the potential toxicity of Amuc_C, the body weights of mice were recorded every three days, but they showed no significant differences between control or treatment groups, suggesting that Amuc_C did not exert substantial toxicity (Figure 3(F)). For further experiments, we harvested the spleens after sacrificing the mice. The mice in the PBS group exhibited evident splenomegaly, whereas the spleens of mice in the Amuc_C group were closer in size to those of healthy mice without tumor inoculation (Figure 3(G)). Additionally, the spleens in the Amuc_C group were significantly lighter than those in the PBS group (Figure 3(H)). These results indicated that Amuc_C had an inhibitory effect on tumor growth and did not pose significant harm to the organisms.

### 3.3. Systemic immune responses activated by Amuc_C increase the frequency of effector T cells and M1 macrophages

**Figure 4.**
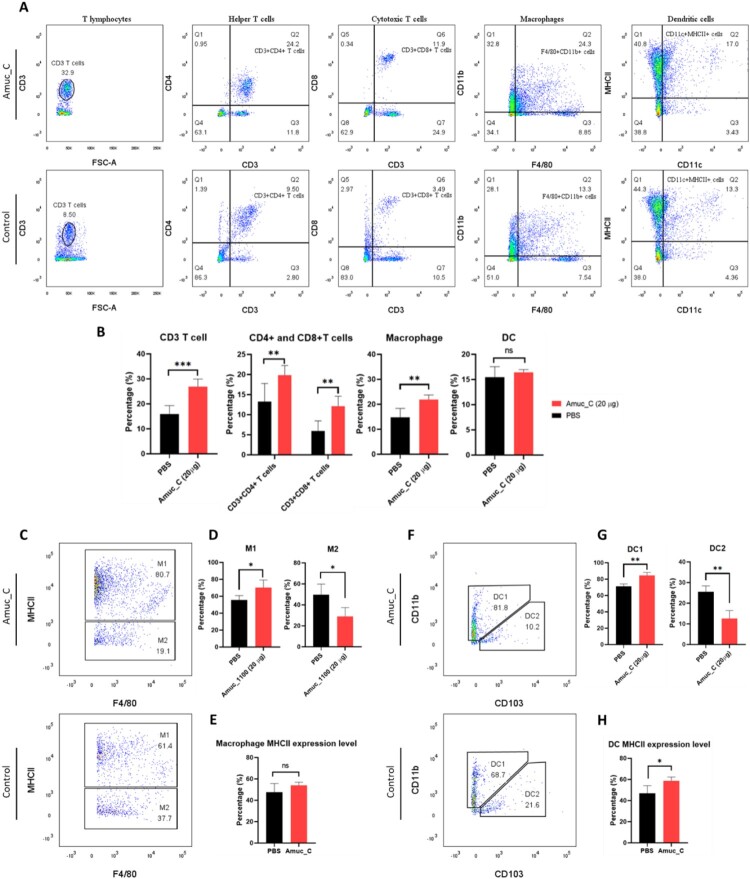
Amuc_C activates T cell and macrophage proliferation. (A) Representative flow cytometry dot plots and (B) bar charts showing several immune cell populations of the splenocytes of control or Amuc_C-treated mice, including T cell lymphocytes, macrophages, and dendritic cells. (C) M1 (upper gate) and M2 (lower gate) macrophages were gated from F4/80+ CD11b + cells, and (D) is the bar charts of the M1 and M2 macrophage percentage of control and Amuc_C group. (E) The MHCII expression of total macrophages. (F) DC1 (upper left gate) and DC2 (bottom right gate) dendritic cells were gated from CD11c + MHCII + cells, and (G) is the bar chart of DC1 and DC2 dendritic cells. Percentage of PBS and Amuc_C group. (H) MHCII expression of total dendritic cells. Data are presented as mean ± SEM. Statistical significance was calculated by unpaired two-tailed Student’s t-test for the comparisons between two groups (ns, not significant; *, *p* < 0.05, **, *p* < 0.01; ***, *p* < 0.001). DC, dendritic cell; MHCII, class II of major histocompatibility complex.

To uncover how Amuc_C impacts the systemic immune system and provokes tumor-inhibitory effects, we conducted flow cytometry to analyze the immune cell composition in the spleens of mice (Figure 4(A)). Significant increases in CD3+ T cells, CD3 + CD4 + helper T cells, CD3 + CD8 + cytotoxic T cells, and macrophages in the Amuc_C group were found; however, no significant changes were identified in DCs (Figure 4(B)). Based on MHCII expressions, macrophages were subclassified into M1 and M2 types (Figure 4(C)). The Amuc_C group exhibited a significantly higher percentage of M1 macrophages and a lower percentage of M2 macrophages (Figure 4(D)). Notably, although the overall percentages of DCs were similar, we found a significantly higher proportion of type 1 DCs (DC1) and diminished expressions of type 2 DCs (DC2) in the Amuc_C group (Figure 4(F and G)). To address the potential reduction in MHCII expression on APCs due to tumors, we separately analyzed the MHCII expression levels on macrophages and DCs. No significant difference in the MHCII expression levels was found in macrophages, but increased levels of MHCII were identified in DCs (Figure 4(E and H)). These findings suggested that Amuc_C administration could increase the expression levels of T cells and macrophages, predominantly M1 macrophages. Furthermore, Amuc_C activated the DCs by triggering MHCII and facilitating DC1 expressions.

### 3.4. Amuc_C increases tumor-infiltrating lymphocytes and M1 macrophages within the tumor microenvironment

**Figure 5.**
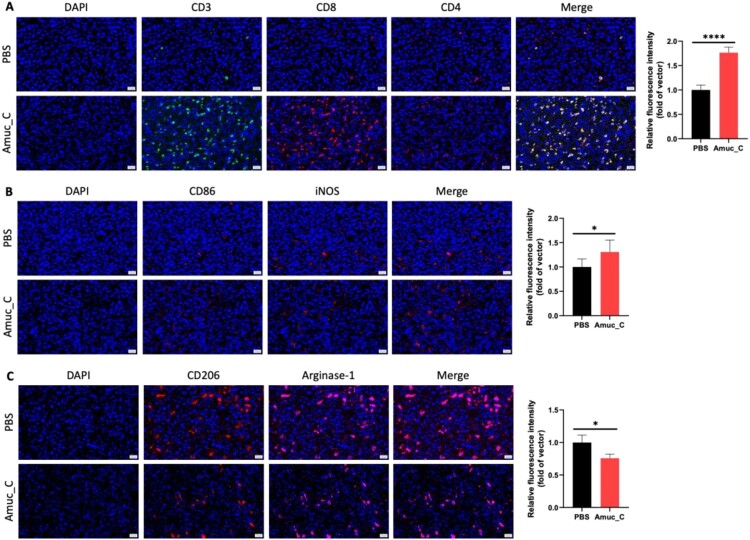
The immunofluorescence staining of CT26 tumors harvested from control and Amuc_C-treated groups. (A) T cell panel. CD3, CD8, and CD4 were stained in green, red, and pink; (B) M1 macrophage panel. CD86 and iNOS were stained pink and red; (C) M2 macrophage panel. CD206 and arginase-1 (Arg-1) were stained in red and pink. Cell nuclei were stained with DAPI. (scale bar = 20 μm). Images were quantified randomly in five fields at 20x magnification. All data are presented as mean ± SEM. Statistical significance was calculated by one-way ANOVA with the Tukey test for the comparisons between all groups (ns, not significant; *, *p* < 0.05; ****, *p* < 0.0001).

Amuc_C effectively activated systemic immune responses; we then assessed whether Amuc_C also increased TILs within tumors. A significant increase in CD3+ T cells and CD8 + cytotoxic T cells in the Amuc_C group was found. In contrast, no changes were found in the number of CD4+ T cells (Figure 5(A)). Tumors often skew macrophages towards the M2 phenotype to promote tumor growth. Repolarizing macrophages into the M1 phenotype can reduce tumor invasiveness (Van Dalen et al. 2018). Compared to the control group, we found an increasing trend in CD86 + iNOS + M1 macrophages in the Amuc_C group (Figure 5(B)), with a lower density of CD206+ Arginase-1+ M2 macrophages (Figure 5(C)). These results suggest that, in addition to activating the systemic immune system, Amuc_C increased the infiltration of CTLs into the tumor and reduced the density of M2 macrophages within the tumor microenvironment, leading to enhanced anti-cancer effects.

### 3.5 Pro-inflammatory cytokines are induced by Amuc_C, leading to the activation of innate immunity

**Figure 6.**
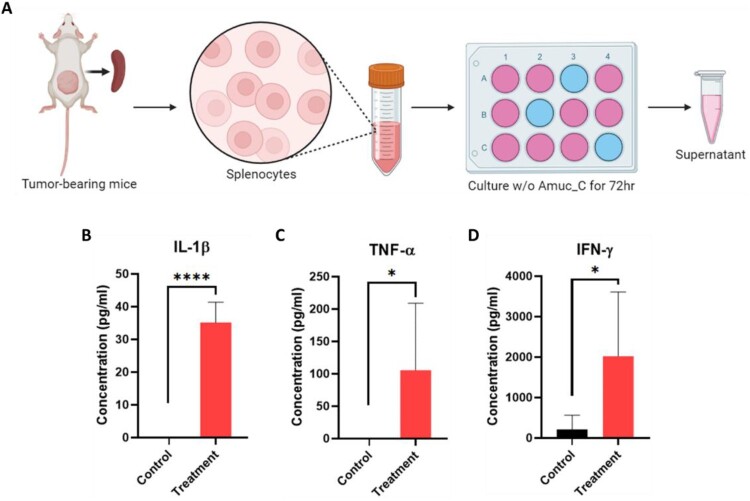
Pro-inflammatory cytokines involved in innate immune activation are induced by Amuc_C *in vivo*. (A) The experiment design of the cytokine ELISA sample preparation. Briefly, spleens were harvested, and the erythrocytes were depleted by ACK lysis buffer. The splenocytes (4 × 10^6^ cells) from each mouse were then seeded into a 12-well tissue culture plate in different wells. The control groups were cultured with PBS, and Amuc_C groups were treated with 10 μg/ml Amuc_C for 72 h, respectively. Finally, the supernatant was collected for cytokine ELISA analysis. (B) Bar charts indicating cytokine levels of IL-1β, TNF-α, and IFN-γ in duplicate (n = 4-5). Data are presented as mean ± SEM. Statistical significance was calculated by unpaired Student’s t-test for the comparisons between two groups (ns, not significant; *, *p* < 0.05; ****, *p* < 0.0001).

To verify whether Amuc_C could activate Th1 immune responses, splenocytes were sampled from untreated tumor-bearing mice with or without the incubation of Amuc_C for 72 h. The cell supernatant was then collected, and pro-inflammatory cytokines, IL-1β, TNF-α, and IFN-γ, were measured (Figure 6(A)). In the control group, few productions of these cytokines were found. However, following stimulation with Amuc_C, splenocytes exhibited significant increases in the secretion of IL-1β, TNF-α, and IFN-γ (Figure 6(B, C, D)). These results indicated that Amuc_C could induce immune cells to secrete higher levels of pro-inflammatory cytokines, thereby eliciting Th1 immune responses against tumors.

### 3.6. Reprogramming of several signaling pathways, including inflammation, immune cell activation, cell cycle arrest, and anti-proliferation, followed the Amuc_C treatment

**Figure 7.**
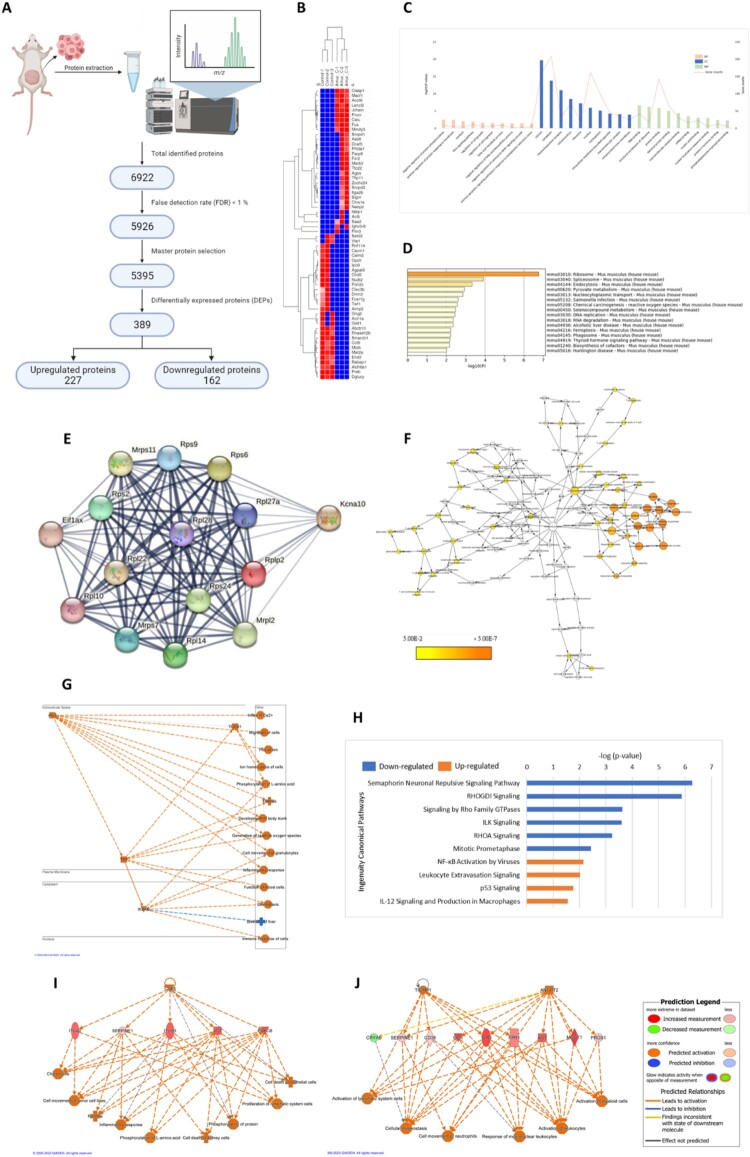
Proteomics and DEP analysis of the Amuc_C-treated CT26 tumor. (A) Pretreated proteins of the tumor masses from the control group and the Amuc_C group were submitted to LC/MS-MS for label-free protein quantification. A total of 6922 proteins were identified and applied for further analysis. (B) Heatmap of representative DEPs of tumors from the control and the Amuc_C group created by the Morpheus network. (C) A representative bar chart of GO term analysis with ten BP, CC, and MF, while (D) is the top 10 KEGG enrichment pathways. All bars represent -log10 (*p*-value). (E) The most important PPI network of DEPs is identified by the MCODE plugin and illustrated by the StringApp plugin in the Cytoscape software. (F) Hub genes for biologucal functions were analyzed using the BiNGO plugin in Cytoscape software. The color bar from yellow to orange indicates the *p*-value from 5.0E-2–5.0E-7. (G) A graphical summary of upstream regulators, related diseases, and functions, as well as (H) representative canonical pathways, were identified by the QIAGEN IPA. The bar represents –log10 (*p*-value) and was shown with a color scheme from upregulation (orange) to downregulation (blue). (I) The mechanistic network of CDK6 and (J) TICAM1 and ANGPT2 were predicted as the most important upstream regulators in epithelial cell death and leukocyte activation by the IPA. The green color indicates decreased expression, while orange represents increased expression; the grey color means the effects were not predicted. BP, biological processes; CC, cellular component; DEP, differentially expressed protein; GO, gene ontology; IPA, ingenuity pathway analysis; KEGG, Kyoto encyclopedia of genes and genomes; LC-MS/MS, liquid chromatography-tandem mass spectrometry; MF, molecular function; PPI, protein-protein interaction.

To further elucidate the mechanism of action of Amuc_C, we extracted proteins from the tumor and conducted LC-MS/MS analysis to examine protein expression patterns. Following initial screening, we identified 389 differential expression proteins (DEPs), comprising 227 upregulated proteins and 162 downregulated proteins (Figure 7(A)). Hierarchical clustering analysis of the genes corresponding to the significantly differentially expressed proteins revealed distinct differences in protein expression between the Amuc_C-treated group and the control group (Figure 7(B)).

To comprehend the functions of these proteins, we employed GO term and enriched KEGG pathway analysis for the DEPs (Figure 7(C,D)). Key biological processes included cell cycle, positive regulation of protein targeting to the membrane, and cytolysis, while the most enriched pathways encompassed pyruvate metabolism, ferroptosis, and biosynthesis of cofactors. To identify the central proteins among these DEPs, we used STRING to obtain protein – protein interaction (PPI) data and imported the PPI network into Cytoscape for further analysis and visualization. Utilizing the MCODE plugin, we identified the highest-ranked cluster, composed of EIF1AX, RPS2, RPL14, RPL28, KCNA10, RPS9, RPL22, MRPS7, RPL10, RPS6 (seed, the highest scoring node), RPL27A, MRPS11, MRPL2, RPLP2, and RPS24 genes, in the network (Figure 7(E) and Table S1).

To explore the overrepresented core biological functions within these hub genes, the BiNGO plugin was employed for analysis (Figure 7(F)). Several biological processes related to T cell differentiation and proliferation, leukocyte differentiation, and the G1/S transition checkpoint were identified. These findings suggest that Amuc_C administration may be associated with regulating immune responses and the mitotic cell cycle. To obtain a more comprehensive understanding, all identified proteins were imported into another bioinformatics application and database, QIAGEN Ingenuity Pathway Analysis (IPA). The graphical summary from IPA revealed that upregulated AGT, TGFB1, TNF, and IKBKB were predicted to activate immune responses, such as cell movement of granulocytes, inflammatory responses, chemotaxis, and the immune response of cells (Figure 7(G)). Upregulation of NF-κB, p53 signaling, and several immune cell-related pathways was observed from the canonical pathways, while downregulation of pathways related to mitosis and cell proliferation was also identified (Figure 7(H)). Regulator effect analysis was conducted to explore additional molecular mechanisms possibly related to the tumor-inhibiting effects of Amuc_C. For cell death of epithelial cells, inflammatory response, and proliferation of lymphatic system cells, CDK6 was predicted to be the top regulator, and both TICAM1 and ANGPT2 were associated with leukocyte activation (Figure 7(I, J) and Table S2).

Taken together, these bioinformatic results indicated that the anti-cancer function of Amuc_C primarily stems from its ability to induce inflammatory responses, activating subsequent immune cell activation, as well as causing cell cycle arrest (G1/S) and inhibiting cell proliferation caused by upregulation of p53 and downregulation of the Rho family pathway. Additionally, CDK6, TICAM1, and ANGPT2 are potential crucial upstream regulatory factors contributing to these effects.

## 4. Discussion

In recent years, *A. muciniphila* (AKK) has garnered significant interest among researchers for its potential therapeutic effects on neoplasms (Derosa et al. 2022). Numerous studies demonstrated the cancer-inhibitory potential of Amuc_1100, a peptide derived from *A. muciniphila* (Gu et al. 2021). However, limited research has focused on the functional regions of this peptide. Our data demonstrate the efficacy of using the C-terminal of Amuc_1100 as a novel TLR2 agonist, which proved highly effective in inhibiting the progression of CRCs. Administration of Amuc_C effectively provoked robust anti-tumor immune responses and significantly suppressed tumor growth.

Several studies have gradually provided evidence supporting the link between IBD and an increased risk of CRCs (Sato et al. 2023), emphasizing the importance of monitoring and managing these conditions to mitigate potential cancer risks. Dysbiosis, or an imbalance in gut bacteria, contributes to inflammation, especially IBD, and may predispose patients to neoplastic changes in the intestinal tracts (Yu 2018). These findings highlight that the inflammatory environment created by IBD can promote tumorigenesis (Terzić et al. 2010; Zhao et al. 2021). Because patients with IBDs exhibit a decrease in beneficial gut bacteria (Ahmadi et al. 2025), an imbalance in the gastrointestinal microbiota may lead to chronic inflammation, resulting in CRCs. Furthermore, we also found that Amuc_C suppressed tumor growth, especially within two weeks (Figure 3(A)). These findings suggested that Amuc_C was suitable for consumption in the early stages of cancer to unleash its robust therapeutic effects. Incorporating Amuc_C as a regular nutritional supplement may be a viable approach for individuals to benefit from its cancer-inhibitory properties. Therefore, this effective substance offers options for patients suffering from CRC and provides treatment for those with IBD, as it may prevent carcinogenesis or serve as a daily nutritional supplement.

Many TLR2 agonists have become adjuvant antigens in vaccine development based on their properties of promoting APC maturation and balancing the activation of different Th-cell responses (Pavot et al. 2014; Luchner et al. 2021). While TLR activation may often lead to cytokine storm (Seya et al. 2015), several preclinical studies have reported that TLR2 agonists are safer than other TLR agonists, but the specific mechanisms have remained unknown (Mantovani et al. 2023). On the other hand, the phenotype switching of TAM is crucial for tumor growth. A previous study indicated that AKK, through the TLR2/NLRP3 signaling pathway, can induce the production of a significant amount of iNOS in M1-type TAM, thereby inhibiting tumor development (Fan et al. 2021b). The current study demonstrated that Amuc_C can effectively induce Th-1 inflammatory responses (Figure 4) and turn cold TME to hot TME (Figure 5), not only enhancing systemic immune responses but also increasing the infiltration of cytotoxic T cells and M1-type macrophages into tumors while reducing the number of M2-type macrophages, which is in line with previous studies (Fan et al. 2021b; Zhang et al. 2023). Furthermore, to clarify the comprehensive changes induced by Amuc_C in tumors, LC-MS/MS and bioinformatic analysis were conducted. In GO term analysis, we found that biological functions such as cytolysis, regulation of cell growth, and regulation of cell migration may be associated with tumor growth inhibition. Furthermore, we also identified increased CD8+ T cells within the tumors (Figure 5(A)) and circulation (Figure 4(A)). Several Th-1 inflammatory cytokines (TNF-α and IFN-γ) were significantly increased after Amuc_C stimulation (Figure 6(C and D)). Therefore, these data might explain the mechanisms of immune activation, the cytolytic effects of which may derive from these cytokines released by CD8+ T cells (Zöphel et al. 2022).

In the hub gene analysis, we identified ribosomal protein S6 (RPS6) as the seed of the cluster. Previous studies have suggested that RPS6 may be associated with regulating cancer cell proliferation, and its phosphorylation is primarily upregulated by the PI3 K/AKT/mTORC1/S6 K pathway (Yi et al. 2021). Elevated expression of RPS6 has been found in many cancers, and research using RPS6-knock-down mouse models has demonstrated a reduction in the proliferation of cancer cell lines (Hagner et al. 2011). In the current study, RPS6 was significantly downregulated in all treatment groups, but it was identified as the highest-scoring node only in the most important cluster of the Amuc_C group. This may indicate that RPS6 could be a key anti-cancer factor of Amuc_1100. The proteomic analysis also revealed that Amuc_C facilitated inflammatory responses through factors such as AGT, TNF, and IKBKB. In the Amuc_C group, we observed upregulation of cell cycle arrest. Furthermore, the upregulation of the p53 signaling pathway, a crucial tumor suppressor, was observed. Research has shown that p53 can regulate ferroptosis and induce the cell death of cancer cells (Wang et al. 2020b), which our findings echo (Figure 7(D)). A recent study also reported that another protein from *A. muciniphila* (Amuc_1434) can disrupt the cell cycle by upregulating the p53 signaling pathway in human CRC cell lines (Luo et al. 2022). These findings could strengthen our results, indicating that Amuc_C might possess similar anti-tumor efficacies, but we believe it is more prudent to conduct further studies to elucidate these underlying mechanisms.

CRCs present a challenging disease of neoplasia due to the limited traditional treatment options and the complexity of managing CRCs. Our study reports for the first time on Amuc_C, an extract derived from a commensal bacterium, which can effectively enhance immune responses in animals and successfully inhibit tumor growth. Studies have shown a strong correlation between IBDs and the progression of CRC, and prior research has also suggested that early administration of TLR2 agonists may serve as an effective treatment strategy to impede tumor progression (Millar et al. 2022). This finding aligns with our current medical concept, which advocates for early medical intervention in chronic inflammation to prevent cancer development. Given the urgent demand for effective immunotherapies in veterinary medicine, our research offers a potential treatment for CRC. In the future, we also aim to apply this immune modulator in treating various chronic inflammatory diseases in humans and/or as an effective agent against different types of cancers.

## Data Availability

The data presented in this study are available on request from the corresponding author.
